# A kind of planar waveguides for cheating the high-speed digital signals into misidentifying the characteristic impedance

**DOI:** 10.1038/s41598-023-41320-0

**Published:** 2023-08-28

**Authors:** Chia Ho Wu, Zhenyu Qian, Wei Wang, Song Tsuen Peng, Chengyang Liu, Jianqi Shen, Donghua Ni, Guoqiang Ye, Fang He, Xiaolong Wang, Linfang Shen, Qichao Li

**Affiliations:** 1 Department of Applied Physics, Zhejiang University of Technology, Hangzhou, 310023 China; 2 Department of Electrical Engineering, National Yang Ming Chiao Tung University, Hsinchu, 30012 Taiwan; 3 Centre for Optical and Electromagnetic Research, College of Optical Science and Engineering, East Building No. 5, Zijingang Campus, Zhejiang University, Hangzhou, 310058 China; 4 Zhejiang Zhaolong Interconnect Technology Co., Ltd, Deqing, Huzhou, 313200 China; 5 Key Laboratory of Quantum Precision Measurement of Zhejiang Province, College of Science, Zhejiang University of Technology, Hangzhou, 310023 China

**Keywords:** Energy science and technology, Engineering, Materials science

## Abstract

Since a planar periodic transmission line can suppress drastically the electromagnetic coupling, it would be advantageous to use such a kind of transmission lines in solving the problem of miniaturization of circuit area. By adjusting the lattice constants and geometric parameters of periodic microstrip lines, a time domain characteristic impedance that is the same as that of conventional microstrip lines (CMLs) can be achieved. Such periodic microstrip lines can therefore be used to trick high-speed digital signals, causing a digital signal to misjudge the time domain characteristic impedance of the transmission lines. The theoretical analysis has been verified by our experimental measurement results. Besides, a specific expression for the characteristic impedance of lossless periodic artificial materials is deduced by a circuit model and a standard of misidentification for the characteristic impedance of periodic microstrip lines is given for the digital signals.

As is known, when a digital circuit is not high in its transmission speed, a digital signal rises for a relatively long time, and this makes it relatively easy to reduce electric circuit area. If, however, a signal speed is fast, the rise time becomes short, and thus, recently, there is a conflict between miniaturization and speedup in design of high-speed digital products due to the shortening of the digital signal rise time of planar high-speed circuit products. Besides, such a shortened digital signal rise time also leads to severe electromagnetic interference, e.g., the crosstalk phenomenon exists in various regions of circuit boards. In general, the ways to reduce the circuit signal crosstalk include the following schemes: enlarging the circuit area, increasing the line interval, reducing the signal speed or lengthening the rise time. Therefore, numerous researchers have worked on developing different effective methods to isolate the crosstalk between high-speed circuits successively for a few decades^1–4^.

In this work, we shall suggest a new scenario for reducing signal crosstalks with a periodic microstrip line structure. As is well known, when a grounded guard trace is introduced into between two parallel microstrip lines, some electric lines of force in signal microstrip lines can be attracted to their grounded guard trace, and then the electromagnetic interference between two microstrip lines can be suppressed efficiently. For this reason, this grounded guard trace for isolating the electromagnetic interference has been used in many commercial products extensively. In order to study the performance of the grounded guard trace for isolating the electromagnetic interference, the authors in Refs.^1–4^ employed electromagnetic numerical methods and experimental technology in resolving the problem of the electromagnetic interaction between grounded guard traces and adjacent microstrip lines on PCBs. However, such grounded guard traces are not completely perfect. For example, when the spacing of adjacent ground holes in a guard trace is sufficiently large, there is resonant coupling between two signal transmission lines^5^, and so the effect of the grounded guard trace on isolating the electromagnetic interference is not significant as the signal frequency increases^6^. In addition, when a grounded guard trace is used to isolate the electromagnetic interference between adjacent microstrip lines, the spacing of two microstrip lines needs to be enlarged in order to hold the grounded guard trace, so that the grounded guard trace is quite adverse in circuit miniaturization. For this reason, researchers began to look for brand new schemes to substitute the grounded guard traces. For example, Lee et al.^7^ and Jiang et al.^8^ have utilized stub-alternated microstrip lines to reduce the electromagnetic interference, including the elimination of far-end crosstalk by increasing the mutual capacitance ratio and reducing the mutual inductance ratio of two adjacent microstrip lines.

In addition to the available isolation schemes, the occurrence of a brand-new domain provides another thinking of isolating the electromagnetic interference between microstrip lines. For example, Pendry et al.^9^ found in theoretical analysis that etching periodically high density holes in a conductor surface would efficiently trap the electromagnetic waves in a certain frequency range. They intended to use this physical phenomenon in actual circuits, especially to suppress the electromagnetic interference between adjacent transmission lines. It was indicated in Refs.^10–14^ that if periodic slots were etched along the edges of microstrip lines, the theoretical analysis and experimental measurement showed that the microstrip lines with periodic texture etched on the edges could efficiently isolate the near- and far-end crosstalks with adjacent microstrip lines.

In order to accelerate the application of these periodic microstrip lines to actual circuits, a first task is to provide the characteristic impedances and equivalent circuits. In the literature, many researchers have built the circuit models for periodic microstrip lines and extracted the circuit parameters, providing the low frequency characteristic impedances^15,16^, so that the $S$-parameters of such periodic microstrip lines can be calculated by using the SPICE simulator and the performance of the circuit systems can be simulated in combination with other active devices.

In the present work, an equivalent network method is used to obtain the exact expression for the characteristic impedance of lossless transmission lines with periodically modulated capacitances and inductances. These characteristic impedances are closely correlated with the capacitances and inductances of the unit cells in the periodic structure, and therefore, the capacitances and inductances in these unit cells can be extracted accurately by quasi-static techniques. In order to obtain the same low-frequency characteristic impedance as that of CML, the lattice constant and the width of the periodic microstrip line will be adjusted. For the microstrip line with periodic slots, the instantaneous impedance variation with time can be detected by using the voltage step-function signal with rise time $R_{{\text{T}}}$. However, it can be shown in numerical result that when the lattice constant becomes smaller to some extent, the instantaneous impedance mutation with time in periodic slots cannot be identified by a voltage step-function signal with rise time $R_{{\text{T}}}$, that is to say, such a step-function signal of voltage (or high-speed digital signals) fails to detect the difference between a periodic microstrip line and a CML. This enables the application of periodic microstrip lines in “deceiving” the digital signals. Since the periodic microstrip lines can efficiently suppress the electromagnetic interference between adjacent microstrip lines, all of CMLs can be replaced by such periodic microstrip lines in high-frequency and high-speed regimes, if the condition under which the digital signals cannot distinguish between CMLs and periodic microstrip lines is fulfilled. In the present work of ours, some actual circuits have been measured by using a time-domain reflectometer, and the measurement result is in good agreement with the theoretical calculation.

## Results

### Equivalent circuit analysis

**Figure 1:**
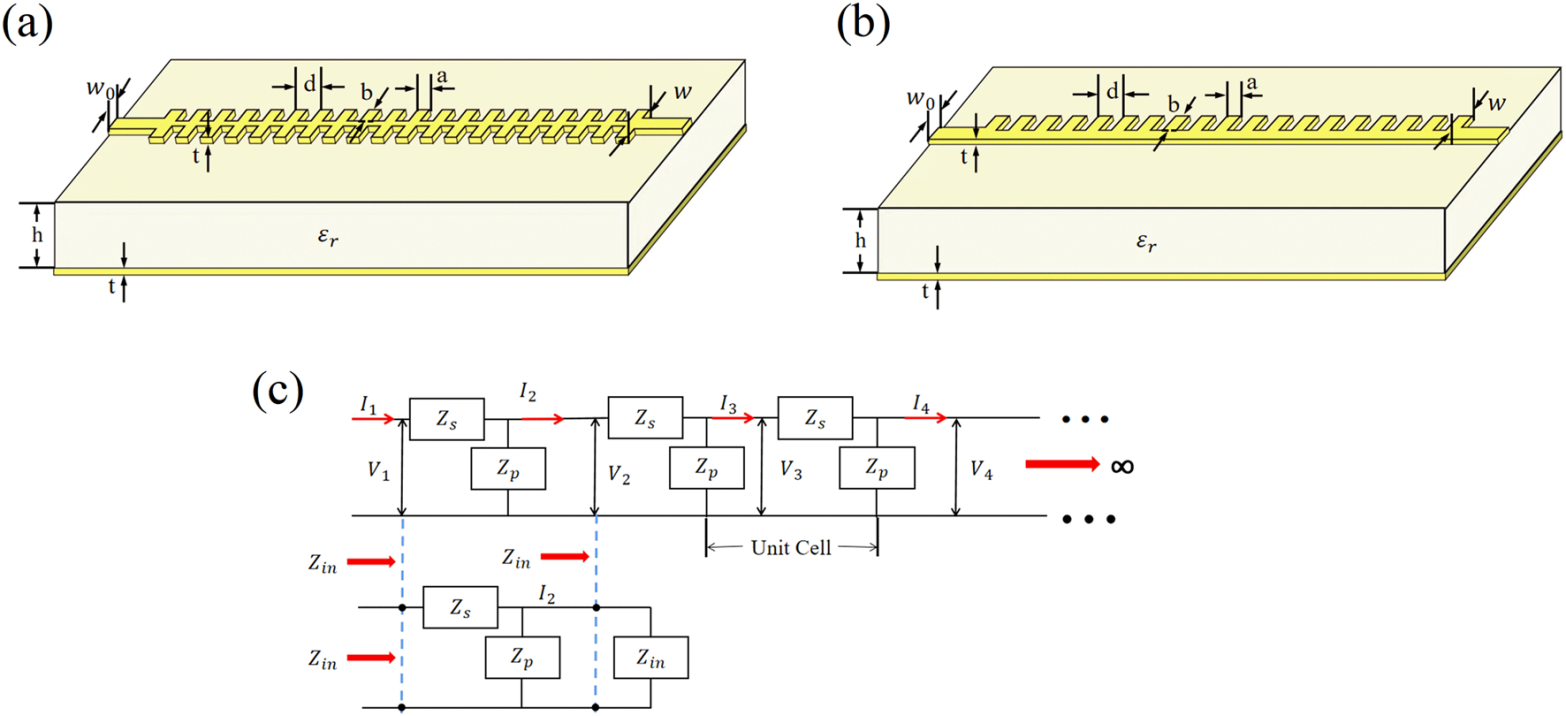
The schematic diagrams of periodic microstrip lines and the circuit model: (**a**) the bilateral periodic microstrip line, (**b**) the unilateral periodic microstrip line, and (**c**) the equivalent circuit diagram of the periodic transmission line.

In this section, we shall present a theoretical principle for our periodic microstrip lines and circuit model. In Fig. 1 we show the schematic diagrams of subwavelength periodic microstrip line (SPML) and the equivalent circuit thereof in this work. In Fig. 1(a) and (b) it is the bilateral and unilateral subwavelength periodic microstrip lines, i.e., BSPML and USPML, respectively. As the edge of the microstrip line is periodically etched, disregarding the conductor resistance and leakage current, the whole microstrip line can be represented by an equivalent network which modulates the inductance and capacitance periodically, as shown in Fig. 1(c). The characteristic impedance of the periodic network can be obtained through the basic units of periodic circuits, where an input impedance method is employed. From the left side of the equivalent circuit in Fig. 1(c), the input impedance $Z_{in}$ can be expressed as

1 $$ Z_{in} = Z_{s} + \frac{{Z_{p} Z_{in} }}{{Z_{p} + Z_{in} }} $$

Then the explicit expression for the characteristic impedance $Z_{in}$ can be given by

2 $$ Z_{in}^{\left( \pm \right)} = \frac{1}{2}\left[ {Z_{s} \pm \sqrt {Z_{s} {(}Z_{s} + {4}Z_{p} {)}} } \right] = \sqrt {Z_{s} Z_{p} } \left[ {\frac{1}{2}\sqrt {Z_{s} Y_{p} } \pm \sqrt {\frac{1}{4}Z_{s} Y_{p} + 1} } \right] $$

where $Z_{s}$ is the inductive impedance $\left( {Z{}_{s} = j\omega L} \right)$ and $Y{}_{p}$ is the capacitive admittance $\left( {Y{}_{p} = j\omega C} \right)$. The resonance frequency of the present $LC$ circuit is defined as

3 $$ \omega_{o} = \sqrt{\frac{1}{LC}}  $$

The square root of multiplication of $Z_{s}$ and $Z_{p}$ is given by

4 $$ Z_{o} = \sqrt{\frac{L}{C}}  = \sqrt {{\text{Z}}_{s} Z_{p} } $$

Let us define the parameter $\theta$ through the following relations

5 $$ \frac{1}{2}\sqrt {Z_{s} Y_{p} } = \frac{1}{2}\sqrt {(j\omega L)(j\omega C)} = j\frac{\omega }{{2\omega_{o} }} = j\sin \theta $$

6 $$ 1 + \frac{1}{4}Z_{s} Y_{p} = 1 - \frac{1}{4}\left( {\frac{\omega }{{\omega_{0} }}} \right)^{2} = 1 - \sin^{2} \theta = \cos^{2} \theta $$

The characteristic impedance expression for the periodic circuit given in Fig. 1(c) can be deduced as

7 $$ Z_{in}^{{( \pm {)}}} = \pm Z_{o} [j\sin \theta \pm \cos \theta ] = \pm Z_{o} e^{ \pm j\theta } $$

where $Z_{in}^{( + )}$ and $Z_{in}^{( - )}$ are the forward and backward Bloch characteristic impedances, respectively. Then by using the capacitance and inductance of unit cell of the periodic structure, the characteristic impedance can be calculated under the lossless transmission line condition. It can be found from Eq. (7) that the characteristic impedance of the periodic microstrip line differs from that of CML only by a phase factor. It is noteworthy that if $\omega < < \omega_{o}$ (i.e., a low-frequency band) or the lattice constant is much smaller than the wavelength, one can have $Z_{in}^{( \pm )} \approx \pm Z_{o}$. According to these equations, all of the relations of the CML are still valid in the low-frequency band. In Fig. 1, the width of the microstrip line is marked as $w$, the thickness of dielectric material is $h$, and the thickness of metal layer is $t$. In this dielectric material, the lattice constant of the periodic structure is $d$ and the dielectric constant is $\varepsilon_{r}$. The width of teeth $a$ is assumed to be half of the lattice constant $d$ in the computing process, and $b$ is the depth of slot.

In our theoretical work, all the circuit parameters were extracted by using COMSOL. In order to verify the theoretical calculation results, the RO4003 circuit board was used herein. In our circuit structure, the dielectric constant of the material is $\varepsilon_{r}$ = 3.37, the thickness of the metal film is $t$ = 0.0175 mm, and the thickness of the dielectric layer is $h$ = 0.508 mm. In order to work out a periodic microstrip line with a specified characteristic impedance, the width of CML is set as $w$ = 1.04 mm intentionally for comparison and the characteristic impedance corresponding to low frequencies is equal to 53.2Ω. Thus, the analysis is relative easy when the time domain signals are used to detect the instantaneous impedance of the transmission lines. The periodic microstrip lines of the three lattice constants have been considered herein, i.e., $d$ = 0.5 mm, 1.0 mm and 2.0 mm. To make the low-frequency characteristic impedance of the periodic microstrip line approximately equal 53.2Ω, for a periodic microstrip line with lattice constant *d* = 0.5 mm, the width of the microstrip line and the depth of the groove must be selected *w* = 1.5392 mm and *b* = 0.2973*w*. Similarly, the structural parameters of the unilateral periodic microstrip line with lattice constant of $d$ = 0.5 mm are $w$ = 1.56 mm and $b$ = 0.6*w*. All the relevant structural dimensions of the periodic microstrip lines are listed in Table 1:

**Table 1:** The geometric dimensions of the subwavelength period microstrip lines whose characteristic impedance approaches that of the conventional microstrip line at low frequencies.

Structure	*d* (mm)	*w* (mm)	*b* (mm)
BSPML(bilateral)	0.5	1.5392	0.4576
	1.0	1.5496	0.4627
	2.0	1.5600	0.4680
USPML(unilateral)	0.5	1.5600	0.9360
	1.0	1.5912	0.9671
	2.0	1.5912	0.9568

**Figure 2:**
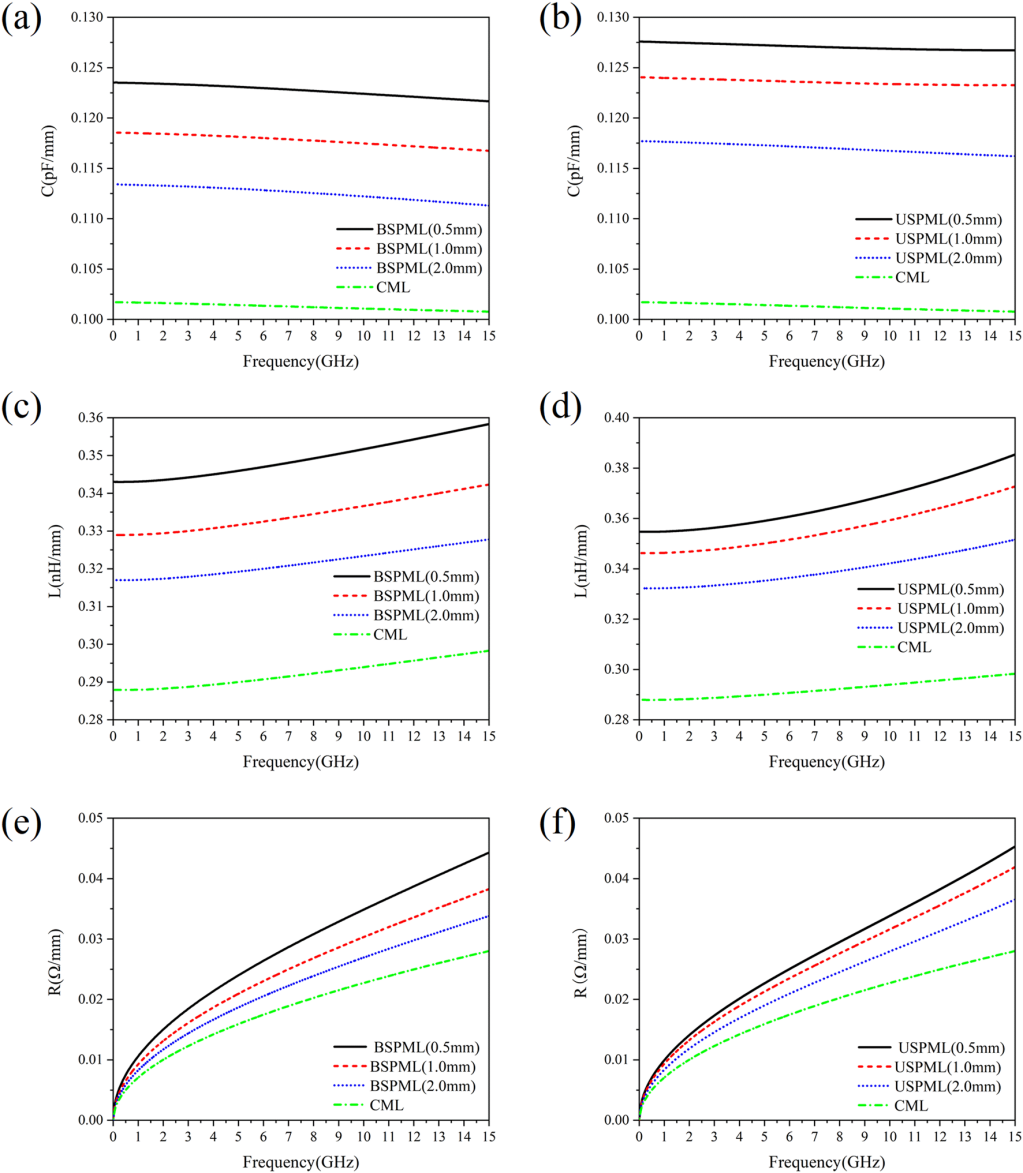
The calculation result of the capacitance and inductance per unit length of the SPMLs: (**a**) the variation of capacitance of the bilateral periodic microstrip lines with frequency, (**b**) the variation of capacitance of the unilateral periodic microstrip lines with frequency, (**c**) the frequency-dependent behavior of the inductance of the bilateral periodic microstrip lines, (**d**) the frequency-dependent behavior of the inductance of the unilateral periodic microstrip lines, (**e**) the per unit length resistance of bilateral subwavelength periodic microstrip lines, and (**f**) the per unit length resistance of unilateral subwavelength periodic microstrip lines.

In order to calculate the characteristic impedance modulus $Z_{0}$, one can be referred to the method provided in Refs.^15,16^, where the capacitance and inductance of the unit length of the periodic microstrip lines can be extracted from the Maxwell equations. The numerical result of the capacitance and inductance per unit length of the SPMLs selected in this paper is shown in Fig. 2. The variation of the capacitance per unit length (with frequency) of BSPMLs and USPMLs in lattice constant of $d$ = 0.5 mm, 1.0 mm and 2.0 mm, respectively, is given in Fig. 2(a) and (b). (It is evident that the capacitance per unit length decreases slowly when the frequency increases, while the value of the capacitance decreases with the increase of the lattice constant.) Note that the main interest of high-speed circuit system is the calculation result of the capacitance and inductance per unit length at low frequencies. At the working frequency *f* = 0.05 GHz, taking the lattice constant *d* = 0.5 mm as an example, the unit length capacitance of BSPML is *C* = 0.12352 pF/mm, while for USPML, *C* = 0.12759 pF/mm, and the capacitance per unit length for CML is 0.10171 pF/mm. In Fig. 2(c) and (d), we show the calculation result of the unit length inductance of BSPML and USPML. For the periodic microstrip lines, the inductance per unit length increases slowly with frequency, and with the decrease of the lattice constant, it helps to increase the self-inductance of periodic microstrip lines. Also at the working frequency $f$ = 0.05 GHz, for the case of lattice constant $d$ = 0.5 mm, the unit length inductance of BSPML $L$ = 0.34301 nH/mm, while for the USPML, $L$ = 0.3547 nH/mm and for the inductance per unit length of CML, $L$ = 0.28793 nH/mm. Then by using the capacitance and inductance per unit length, the characteristic impedance modulus $Z_{0}$ can be obtained from Eq. (4). To enable the circuit model to provide an accurate *S*-parameters calculation result, the resistance $R$ value of metallic conductors in the microstrip line can be obtained by the perturbation method^15^ and the conductance $G$ can be calculated by using the relevant equations given in Ref.^17^. Since the subwavelength periodic microstrip lines can efficiently constrain the electromagnetic field and hence isolate the electromagnetic interference between adjacent microstrip lines, it is necessary to consider the skin effect of the metal. According to the literature^18^, the average power dissipated in electromagnetic waves on the surface of a conductor can be obtained by calculating the surface area integral of the magnetic field square:

8 $$ P_{av} = \frac{1}{2}R_{s} \iint\limits_{S} {\left| {H_{t} } \right|^{2} ds} $$

where $R_{s}$ is the surface resistance of the conductor. It can be expressed in terms of the real part of the intrinsic impedance $\eta$ of the conductor, i.e.,

9 $$ R_{s} = {\text{Re}} \left( \eta \right) = {\text{Re}} \left[ {\left( {1 + j} \right)\sqrt {\frac{\omega \mu }{{2\sigma }}} } \right] = \sqrt {\frac{\omega \mu }{{2\sigma }}} = \frac{1}{{\sigma \delta_{s} }} $$

Here *δ*_*s*_ is the skin depth. The resistance of a conductor can be obtained through the following expression

10 $$ P_{av} = \frac{1}{2}R\left| I \right|^{2} $$

The behavior of dependence of the resistance per unit length on the frequency in the subwavelength periodic microstrip lines is shown in Fig. 2(e) and (f), where the numerical result of the resistance of conventional microstrip lines (CMLs) is also included for comparison. Then it can be seen that with the decrease of the lattice constant, the resistance per unit length gradually increases, and is larger than that of the conventional microstrip lines. This is because the subwavelength period microstrip lines can be more efficient in confining the electromagnetic fields than the conventional microstrip lines.

**Figure 3:**
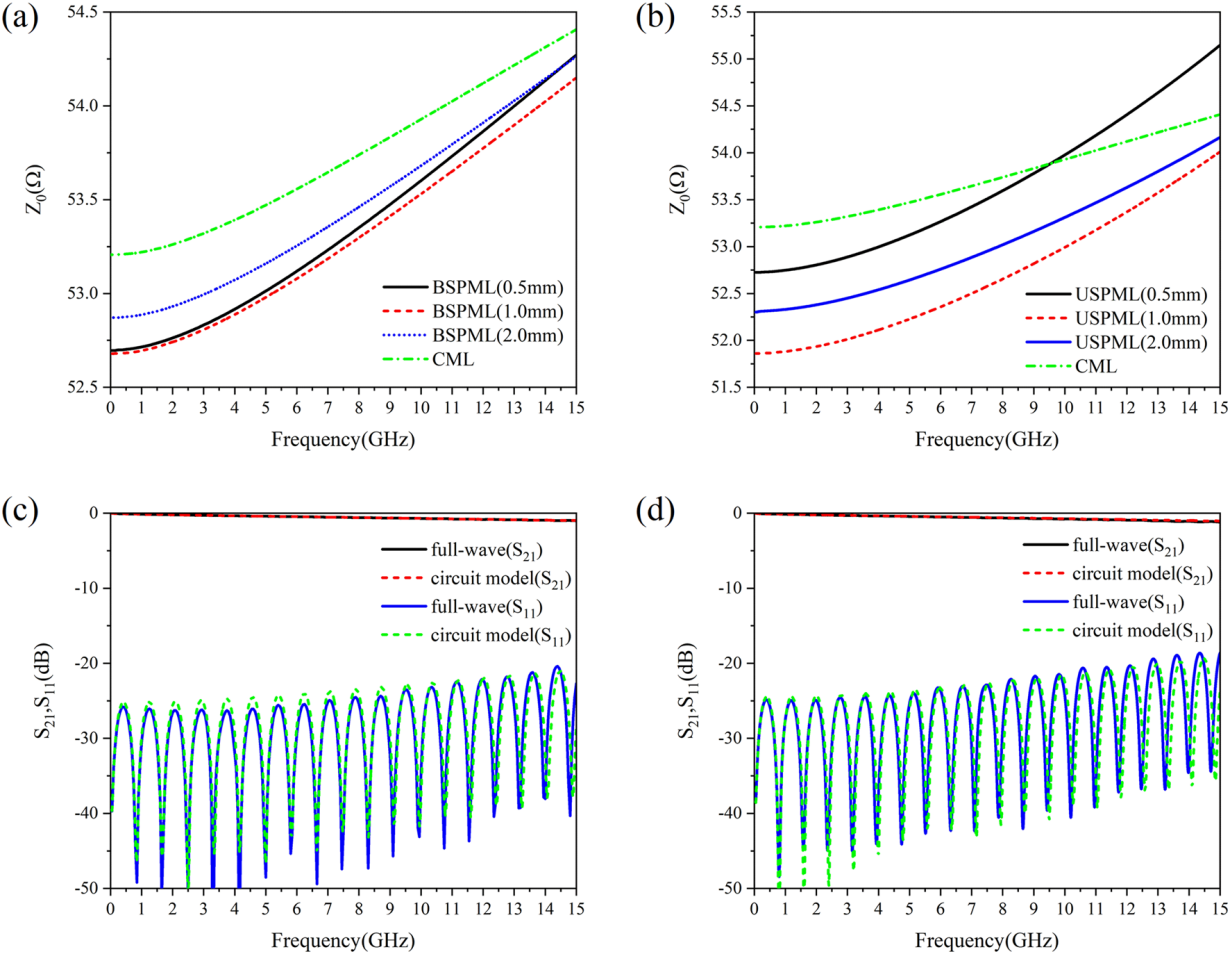
The dispersion behavior of the characteristic impedance modulus $Z_{0}$ and *S*-parameters: (**a**) the variation of characteristic impedance modulus $Z_{0}$ of the bilateral periodic microstrip lines with frequency, (**b**) the variation of characteristic impedance modulus $Z_{0}$ of the unilateral periodic microstrip lines with frequency, (**c**) the frequency-dependent behavior of *S*-parameters of the bilateral periodic microstrip lines, and (**d**) the frequency-dependent behavior of *S*-parameters of the unilateral periodic microstrip lines.

The dispersion behavior of the characteristic impedance modulus $Z_{0}$ and *S*-parameters of the periodic microstrip lines in the numerical simulation is given in Fig. 3. In Fig. 3(a) and (b) we show the variation of $Z_{o}$ with frequency obtained by using the circuit parameters. For the BSPMLs at low frequencies (e.g.,$f$ = 0.05 GHz), the characteristic impedance modulus $Z_{0}$ of lattice constant $d$ = 0.5 mm, 1.0 mm and 2.0 mm is 52.696Ω, 52.680Ω and 52.872Ω, respectively, where the maximum difference from $Z_{0}$ = 53.207Ω of CML is only 0.527Ω. For the USPMLs at low frequencies (e.g.,$f$ = 0.05 GHz), the characteristic impedance modulus $Z_{0}$ of lattice constant $d$ = 0.5 mm, 1.0 mm and 2.0 mm is 52.780Ω, 52.829Ω and 53.129Ω, respectively, where the maximum deviation from $Z_{0}$ = 53.207Ω of CML is only 0.427Ω. It is seen in the numerical calculation that as long as the geometric parameters of SPMLs with different lattice constants are adjusted, the numerical difference of the characteristic impedance modulus $Z_{0}$ between the periodic microstrip line and the CML (with the width of $w$ = 1.04 mm) can be limited within only 0.8%. The *S*-parameters simulation result of BSPML and USPML with lattice constant $d$ = 2.0 mm is shown in Fig. 3(c) and (d), where the solid lines represent the full wave simulation result and the dashed lines denote the *S*-parameters calculated by the circuit model. It can be found that the two results are consistent with each other. Here, the full length of the periodic microstrip line is 10 cm.

As is known, the characteristic impedance of CML or the change in the impedance (resulting from the width variation) of the microstrip line can be detected by importing a voltage step-function signal (with rise time $R_{{\text{T}}}$) into the microstrip line (i.e., Time Domain Reflectometry)^19^. Therefore, when a microstrip line has slots, the instantaneous impedance mutation (in the spatial coordinate axis) can be detected by means of the reflected waves of voltage step-function signals transmitted in the microstrip line, where the instantaneous impedance mutation in the spatial coordinate axis can be represented in time domain. We point out that when a step-function signal passes through a microstrip line with periodically distributed slots, there exists a series of instantaneous impedance peaks along the microstrip line. When the lattice constant decreases, the peaks of instantaneous impedance approach each other in time domain, i.e., these peaks of instantaneous impedance become unidentifiable gradually. Therefore, when the transmission time of a step-function signal in a unit cell of periodic structure is shorter than half of the step-function signal rise time, the reflected waves generated by the unit cells in the periodic structure will be unavoidably unidentifiable (i.e. the reflected waves is covered by the rising edge). Thus, the relation between the minimum lattice constant $d_{\min }$ can be resolvable and the rise time $R_{{\text{T}}}$ that can make the step-function signal misjudge the instantaneous impedance of the periodic microstrip line can be expressed as follows:

11 $$ d_{\min } = \frac{{vR_{T} }}{2} $$

where $v$ is the velocity of the step-function signal in the microstrip line. When the lattice constant $d$ of the periodic microstrip line is smaller than $d_{\min }$, the reflected waves of the voltage step-function signals cannot resolve the impedance mutation induced by two adjacent slots in the periodic structure of the microstrip line. Then in this case, the whole periodic microstrip line can be regarded as a uniform microstrip line with some instantaneous impedances. Taking the circuit board used in this work as an example, for a step-function signal with rise time $R_{{\text{T}}}$ = 30 ps, the value of $d_{\min }$ is 0.2775 mm.

**Figure 4:**
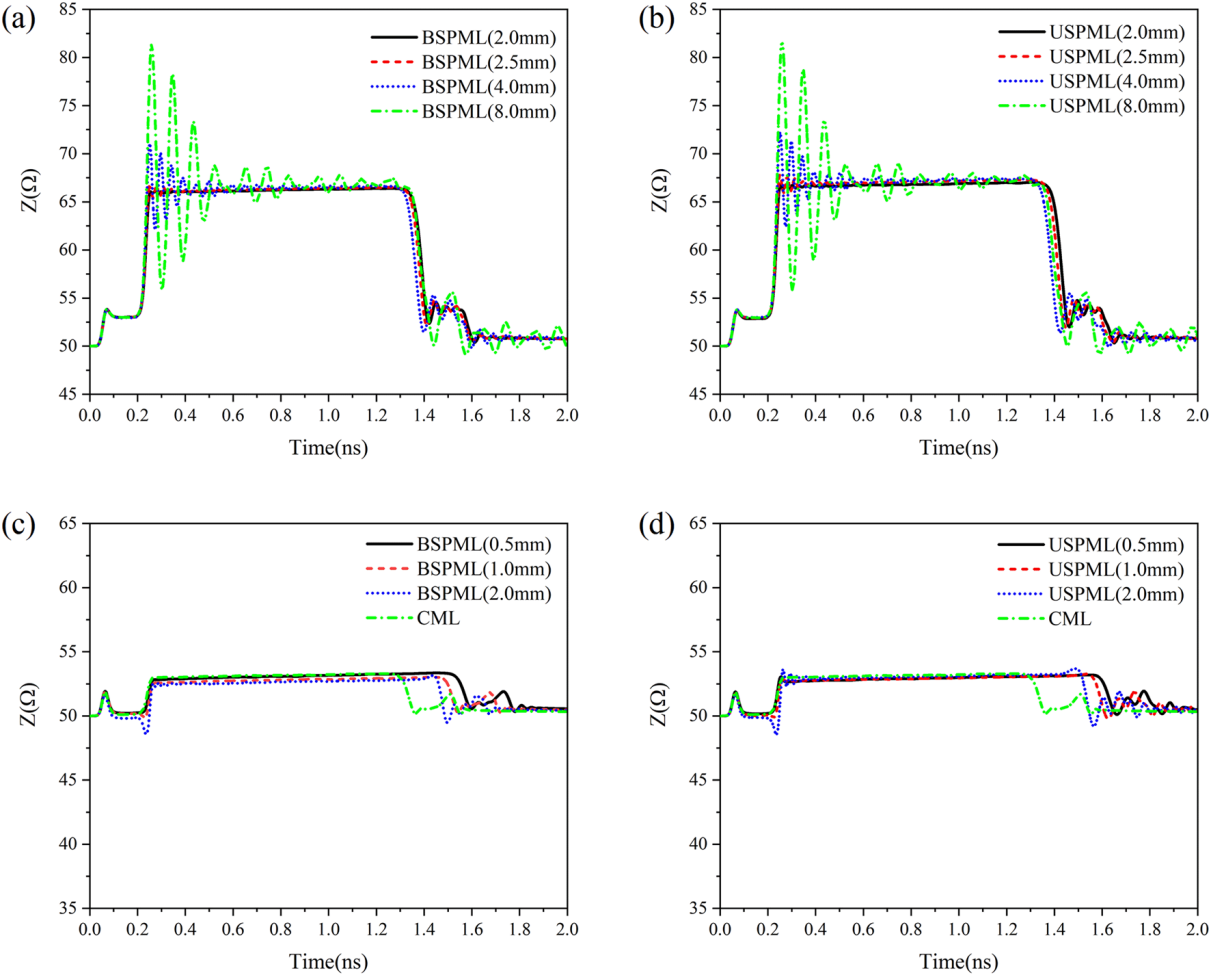
The time dependence of the instantaneous impedance of the periodic microstrip lines by means of the reflected waves of time-domain pulses: (**a**) the calculation result of the instantaneous impedance of the bilateral periodic microstrip lines with lattice constants of 8.0 mm, 4.0 mm, 2.5 mm and 2.0 mm and with line width $w$ = 1.04 mm, (**b**) the calculation result of the instantaneous impedance of the unilateral periodic microstrip lines with lattice constants of 8.0 mm, 4.0 mm, 2.5 mm and 2.0 mm and with line width $w$ = 1.04 mm, (**c**) the instantaneous impedance calculation result of the bilateral periodic microstrip lines with geometry dimensions in Table 1, and (**d**) the instantaneous impedance calculation result of the unilateral periodic microstrip lines with geometric dimensions in Table 1.

**Figure 5:**
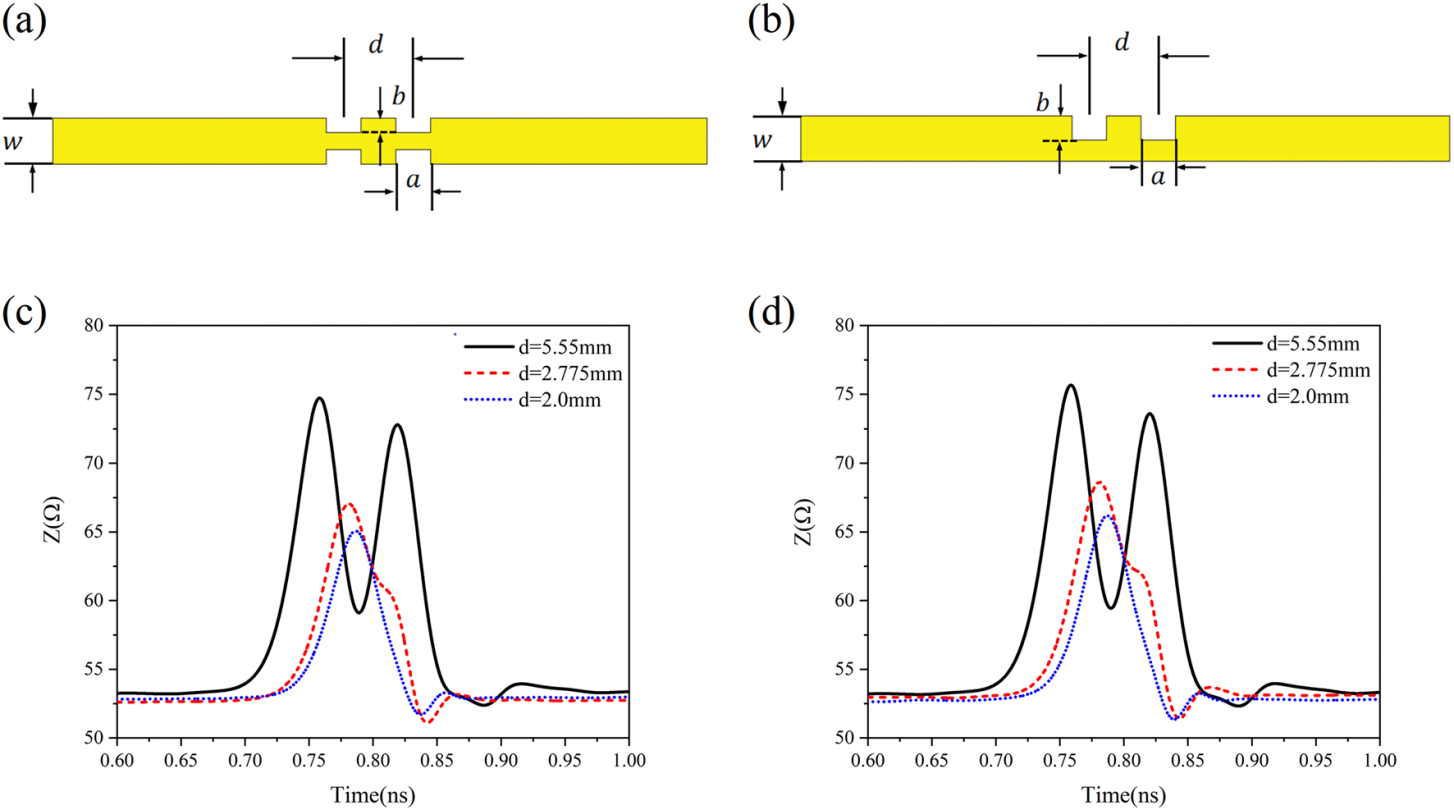
The structures and the numerical result: (**a**) the schematic diagram of two bilateral grooves with center distance $d$ on the microstrip line, (**b**) the schematic diagram of two unilateral grooves with center distance $d$ on the microstrip line, (**c**) the change in the instantaneous impedance of two bilateral grooves when the center distance varies, and (**d**) the change in the instantaneous impedance of two unilateral grooves when the center distance varies.

Now we shall show in Fig. 4 the instantaneous impedance variation (depending on time) of the periodic microstrip lines obtained by using the reflected waves of time-domain pulses. In Fig. 4(a) and (b), the periodic slots with lattice constant $d$ = 8.0 mm, 4.0 mm, 2.5 mm and 2.0 mm were etched in the CML with width $w$ = 1.04 mm and length 10 cm, where the slot depths of BSPMLs and USPMLs are 0.3 $w$ and 0.6 $w$, respectively. The result from the calculation verifies that for the step-function signal with a rise time of $R_{{\text{T}}}$ = 30 ps, when the lattice constant is smaller than $d$ = 2.775 mm, the instantaneous impedance oscillation amplitude exhibits very weak ripples only at the beginning. In Fig. 4(c) and (d), we show the instantaneous impedance variation (depending on time) obtained from the reflected signals after the step-function signal with rise time of 30 ps has been imported into the bilateral and unilateral periodic microstrip lines with lattice constant $d$ = 0.5 mm, 1.0 mm and 2.0 mm, where the widths of the periodic microstrip lines were taken from Table 1. In Ref.^18^ it was indicated that when the instantaneous impedance of a transmission line does not vary with time, it can be regarded as a characteristic impedance. In addition, according to Ref.^19^, when the loss is very small, the result obtained by a time-domain signal reflectometry can be identified as the low-frequency characteristic impedance of the transmission line. The instantaneous impedance of the CML in width of $w$ = 1.04 mm changes between 53.0Ω and 53.26Ω in the time range from $t$ = 0.292 ns to 1.228 ns, and from Fig. 4(c) and (d), the instantaneous impedance of CML in this time interval is almost a horizontal line. Then the median is defined as the characteristic impedance ($Z_{c}$ = 53.1653Ω) for the CML. To make the characteristic impedance of BSPML with lattice constant $d$ = 0.5 mm approximate the CML of width of $w$ = 1.04 mm, the line width and slot depth of the periodic line are $w$ = 1.5392 mm and $b$ = 0.2973$w$, respectively. The time domain analysis shows that the characteristic impedance $Z_{c}$ = 53.1187Ω. In the view of time-domain analysis, the difference between the characteristic impedance designed for the bilateral periodic microstrip line with lattice constant $d$ = 0.5 mm and the characteristic impedance of the conventional microstrip line is only 0.08%. As for the USPML with lattice constant $d$ = 0.5 mm, if the width of the microstrip line is $w$ = 1.56 mm and the slot depth is $b$ = 0.6*w*, the characteristic impedance obtained by using the time domain analysis is $Z_{c}$ = 52.955Ω. Obviously, if a periodic microstrip line with an appropriate width is selected, its characteristic impedance can be close extremely to that of CML, as long as the condition of Eq. (11) is fulfilled. In order to further check the correctness of Eq. (11), a step-function signal with a rise time of 30 ps is considered here to distinguish two grooves continuously distributed along the microstrip lines as shown in Fig. 5(a) and (b). As can be seen from the numerical results, when the interval between two grooves is larger than 2.775 mm, such as $d$ = 5.5 mm, the step-function signal with the rise time of $R_{{\text{T}}}$ = 30 ps can completely distinguish the two grooves, whereas in the case of the interval $d$ = 2.0 mm, the step-function signal cannot distinguish the two grooves. The numerical results relevant to the above property are shown in Fig. 5(c) and (d). As is known, when using the function of the time domain reflectometer, the rise time of the step-function signal is often related intimately to the bandwidth of the signal. In the present work, by using the time domain reflectometer, the relation between the bandwidth of the signal and the rise time of the digital signal is $f_{\max } = 0.5/R_{T}$^20^ (this usually originates from the requirements of the instrument itself), and hence from Eq. (11) we can obtain

12 $$ d_{\min } = \frac{{vR_{T} }}{2} = \frac{v}{{4 \times 0.5/R_{T} }} = \frac{{\lambda_{\min } }}{4} $$

**Figure 6:**
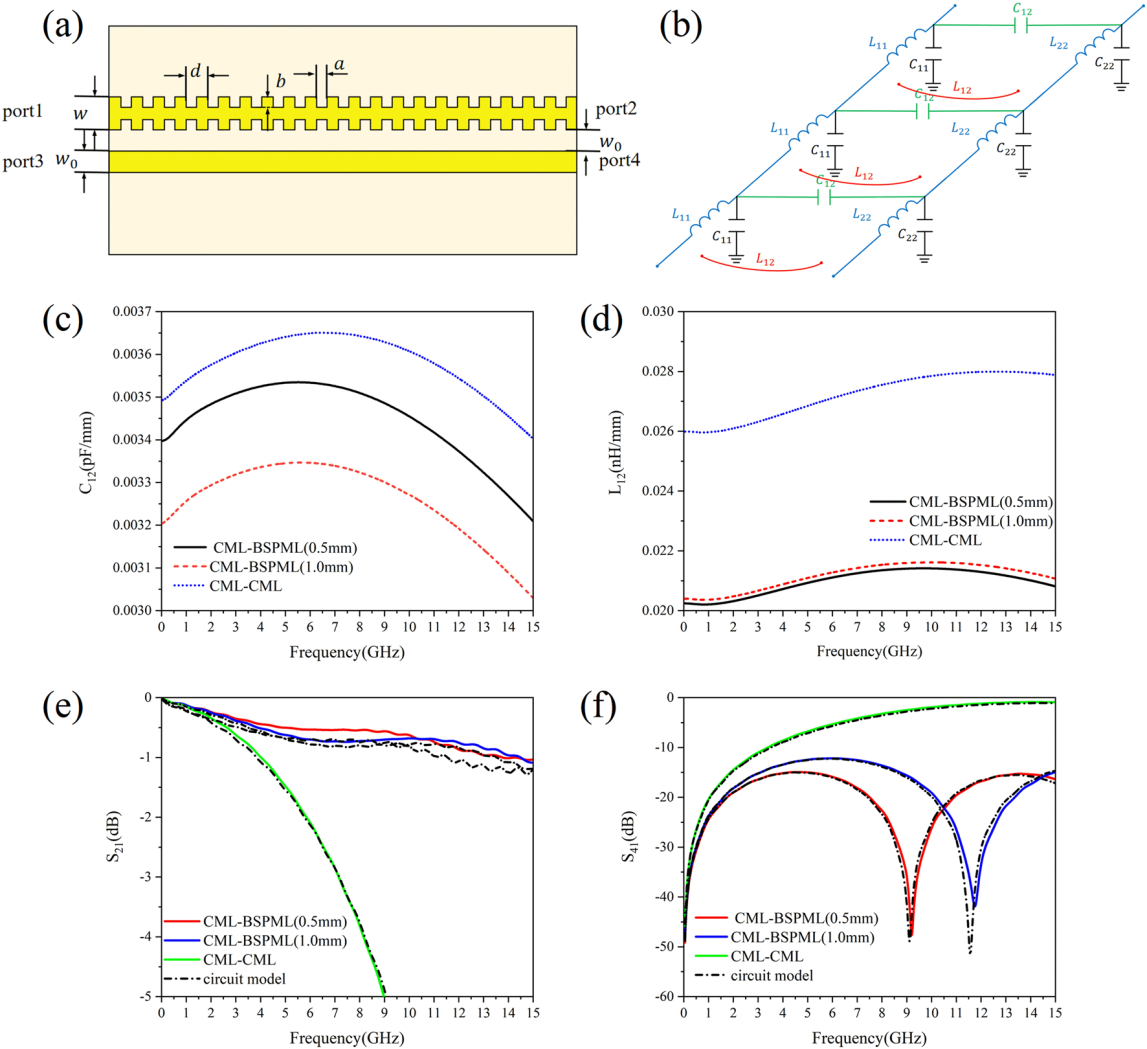
The structure and the numerical results: (**a**) the schematic diagram of coupled circuits composed of a bilateral subwavelength periodic microstrip line and a conventional microstrip line, (**b**) the circuit model of the coupled circuit of a periodic microstrip line and a conventional microstrip line, (**c**) the dependence of the mutual capacitance on frequency, (**d**) the dependence of the mutual inductance on frequency, (**e**) the change in $S_{21}$ depending on frequency, and (**f**) the change in $S_{41}$ depending on frequency.

It can be seen that when the interval between the two grooves is less than a quarter of the shortest wavelength, the step-function signal with the rise time $R_{{\text{T}}}$ cannot distinguish between the adjacent grooves. In order to explore the reasons for the suppression of electromagnetic crosstalk by subwavelength periodic microstrip lines, two 10-cm-long parallel microstrip lines are taken into account in Fig. 6(a), one of which is composed of CML, and the other is the subwavelength bilateral periodic microstrip line as indicated in Table 1. Figure 6(b) is the circuit model of the coupled circuit of a periodic microstrip line and a conventional microstrip line. In order to analyze the coupling strength between the periodic microstrip line and the conventional microstrip line, the variation of mutual capacitance with frequency can be calculated by the capacitance matrix equation

13 $$ \left( {\begin{array}{*{20}c} {Q_{1} } \\ {Q_{2} } \\ \end{array} } \right) = \left( {\begin{array}{*{20}c} {C_{11} + C_{12} } & { - C_{12} } \\ { - C_{12} } & {C_{22} + C_{21} } \\ \end{array} } \right)\left( {\begin{array}{*{20}c} {V_{1} } \\ {V_{2} } \\ \end{array} } \right) $$

The variation of mutual inductance with frequency is calculated by the inductance matrix equation

14 $$ \left( {\begin{array}{*{20}c} {\varphi_{1} } \\ {\varphi_{2} } \\ \end{array} } \right) = \left( {\begin{array}{*{20}c} {L_{11} } & {L_{12} } \\ {L_{21} } & {L_{22} } \\ \end{array} } \right)\left( {\begin{array}{*{20}c} {I_{1} } \\ {I_{2} } \\ \end{array} } \right) $$

We show the frequency-dependent behavior of the mutual capacitance and the mutual inductance in Fig. 6(c) and (d), respectively, where the calculation result for the mutual capacitance and the mutual inductance of two conventional microstrip lines is also included for the sake of comparison. It can be seen that, compared with a system consisting of two conventional microstrip lines, the system consisting of a periodic microstrip line and a conventional microstrip line as presented in Fig. 6(a) has relatively small mutual capacitance and mutual inductance. In order to verify the correctness of these circuit parameters, the *S*-parameters have been calculated by using the circuit model and compared with the numerical results of the full wave simulation. Figure 6(e) and (f) show the change in $S_{21}$ and $S_{41}$, respectively, which depend on frequency. The comparison results show that the two methods agree well with each other. It can be seen that when one of the conventional microstrip lines is replaced by a periodic microstrip line, the numerical result of $S_{41}$ is much smaller than that of the two conventional microstrip lines. This is also the main reason for the suppression of remote crosstalk. In practice, the circuit model in Fig. 1(c) should be considered to be applicable only to the case of very short transmission line segments, and when the distributed parameters are replaced by lumped parameters, a large number of lumped circuits in series are required to replace each small segment of transmission line, as is the case in standard microwave engineering textbooks^18^. For example, the lattice constants of the subwavelength period microstrip lines are taken to be 0.5 mm, 1.0 mm, and 2.0 mm, which are far smaller than the minimum wavelength corresponding to the frequency range under consideration. Therefore, it is appropriate to use such circuit models in the present work.

### Experimental results

**Figure 7:**
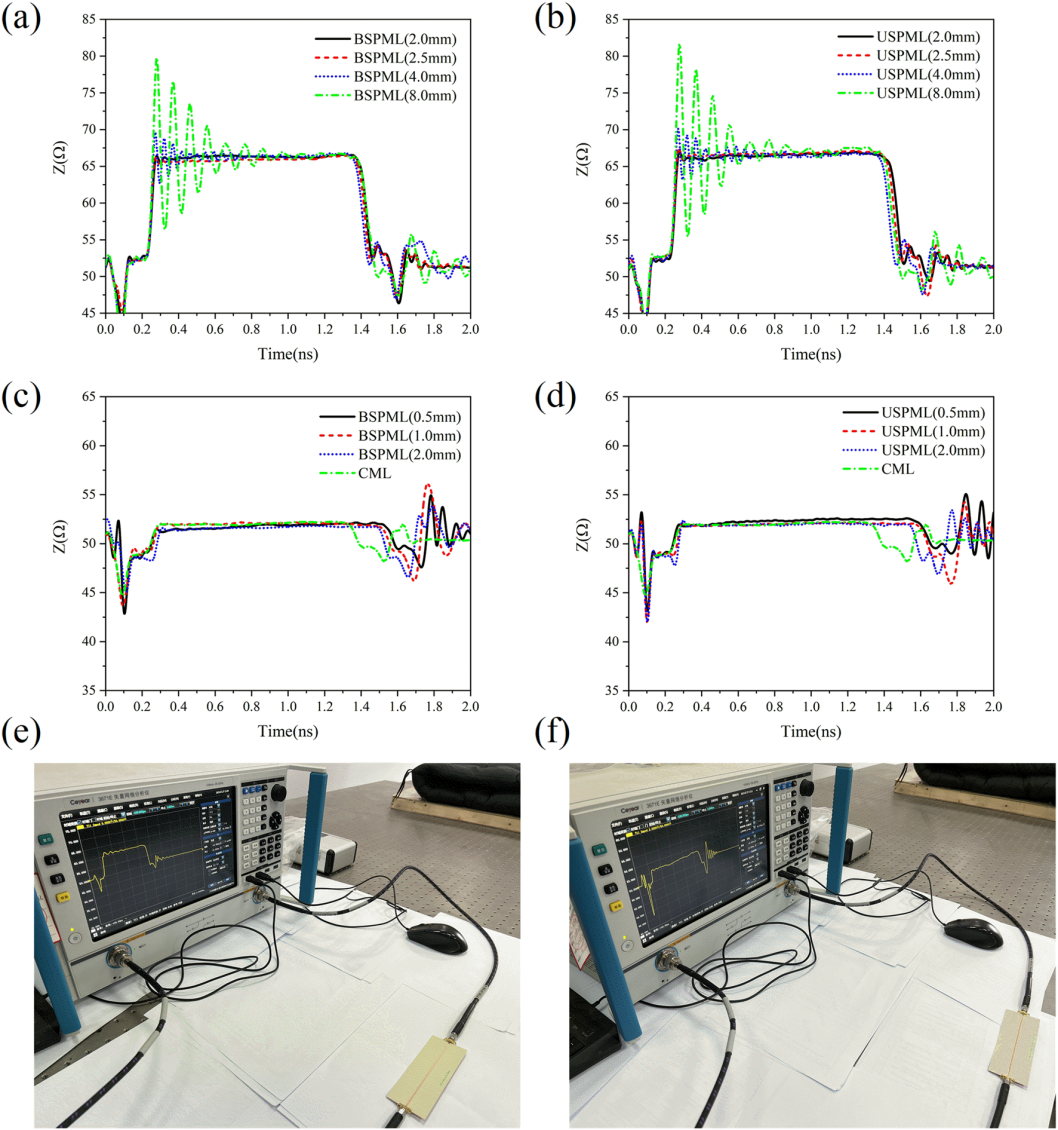
The experimental observation for the evolutional instantaneous impedance: (**a**) the measurement result of the instantaneous impedance of the bilateral periodic microstrip line with line width $w$ = 1.04 mm, (**b**) the measurement result of the instantaneous impedance of the unilateral periodic microstrip lines with line width $w$ = 1.04 mm, (**c**) the instantaneous impedance measurement result of the bilateral periodic microstrip lines with geometric dimensions in Table 1, (**d**) the instantaneous impedance measurement result of the unilateral periodic microstrip lines with geometric dimensions in Table 1, and the photos (**e**), (**f**) represent the instrument for measuring the circuits in our experiment.

In order to verify the accuracy of Eq. (11), a microstrip line of width 1.04 mm and of length 10 cm is selected for designing the unilateral and bilateral periodic microstrip lines with lattice constants $d$ = 8.0 mm, 4.0 mm, 3.0 mm, 2.5 mm and 2.0 mm, respectively. The voltage step-function signals with rise time of 30 ps were imported into the periodic microstrip lines for detecting the characteristic impedance of the periodic microstrip lines. In general, the production process of such a circuit board includes: We can first draw the subwavelength period microstrip lines in accordance with the selected sizes by using the engineering drawing software, require the relevant manufacturers to make the photographic plate of such a circuit board, and then we carry out exposure etching of the circuit of RO4003 in order to complete the production of the circuit board. The measurement result of the instantaneous impedance of the bilateral and unilateral periodic microstrip lines in width of $w$ = 1.04 mm is presented in Fig. 7(a) and (b). It can be seen that the measurement result in Fig. 7(a) and (b) is very consistent with the time-domain numerical result in Fig. 4(a) and (b). The instantaneous impedance (measured by Time Domain step-function signals) of the bilaterally and unilaterally slotted periodic microstrip lines of the sizes given in Table 1 is shown in Fig. 7(c) and (d), where, as predicted by the theoretical analysis, the relation of instantaneous impedance to time is a horizontal line. It can be observed that the measured characteristic impedance value of the CML in width of $w$ = 1.04 mm is $Z_{o}$ = 52.0698Ω, the characteristic impedance of the bilateral periodic microstrip line in width of $w$ = 1.5496 mm with lattice constant $d$ = 1.0 mm is $Z_{o}$ = 52.1298Ω, and the characteristic impedance of the unilateral periodic microstrip line in width of $w$ = 1.5912 mm with lattice constant $d$ = 1.0 mm is $Z_{o}$ = 52.0785Ω. It can be found that the measured instantaneous impedance result of Fig. 7(c) and (d) is consistent with the numerical result of Fig. 4(c) and (d). The impedance measuring instrument is shown in Fig. 7(e) and (f), which is the one for the time-domain reflectometer attached to the network analysis. To measure the instantaneous impedance of a periodic microstrip line, it is necessary to input a step-function signal with a rise time $R_{{\text{T}}}$ to the present microstrip line. The reflection coefficient resulting from the impedance mismatch can be expressed as^21^

15 $$ \Gamma = \frac{{V_{reflected} }}{{V_{incident} }} = \frac{{Z_{DUT} - Z_{0} }}{{Z_{DUT} + Z_{0} }} $$

The impedance $Z_{DUT}$ of the transmission line to be measured can be expressed as

16 $$ Z_{DUT} = {\text{Z}}_{0} \frac{{V_{incident} + V_{reflected} }}{{V_{incident} - V_{reflected} }} $$

where $V_{incident}$ is the incident voltage and $V_{reflected}$ the reflected voltage.

**Figure 8:**
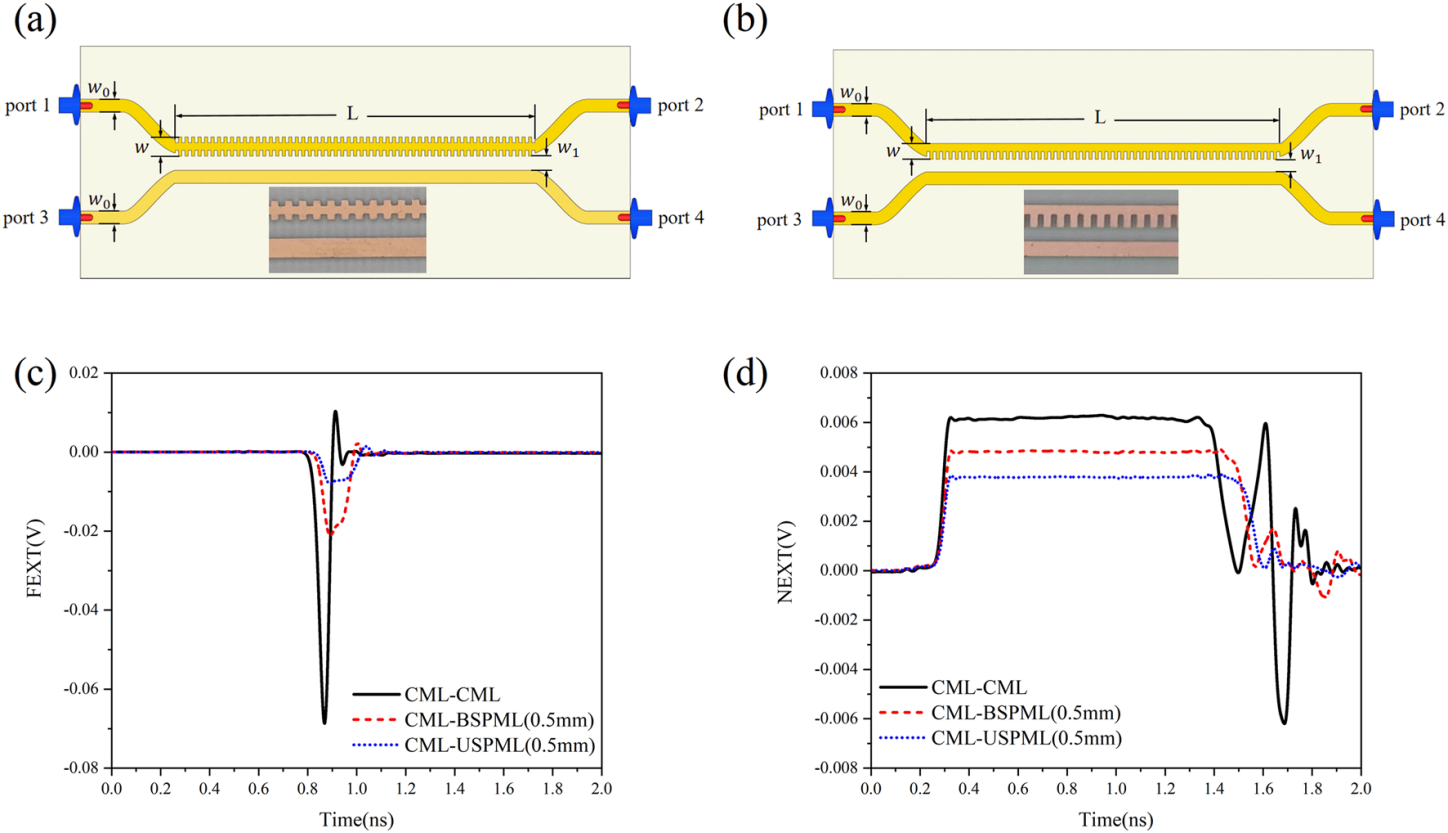
The coupled circuits composed of a bilateral/unilateral periodic microstrip line and a CML: (**a**) the coupled circuit composed of the bilateral periodic microstrip line and the CML, (**b**) the coupled circuit composed of the unilateral periodic microstrip line and the CML, (**c**) the FEXT measurement result of the coupled circuit composed of the periodic microstrip line and the CML, and (**d**) the NEXT measurement result of the coupled circuit composed of the periodic microstrip line and the CML.

Now we shall verify the performance of the periodic microstrip lines for suppressing the far-end crosstalk (FEXT) and near-end crosstalk (NEXT). In this process, the voltage step-function signal with rise time of 30 ps and amplitude of 0.2 V was imported into one port of the periodic microstrip line. The circuits that can be used to measure the time-domain signals are shown in Fig. 8(a) and (b), where the coupled circuit composed of a BSPML and a CML is given in panel (a) and the coupled circuit composed of a USPML and a CML is given in panel (b). The coupled microstrip circuit FEXT and NEXT measurement results are presented in Fig. 8(c) and (d), respectively, where the coupled zone of two parallel microstrip lines is 10 cm and the spacing of the two microstrip lines is 1.04 mm. It can be observed in Fig. 8(c) that the peak value of the far-end crosstalk (FEXT) of the two CMLs is $- 0.06886$ V, i.e., the crosstalk accounts for about 34.33% of the amplitude of the incident signals. If one of the CMLs is replaced by a BSPML with lattice constant of 0.5 mm, the peak value of the FEXT is $- 0.02188$ V, i.e., the crosstalk accounts for about 10.59% of the amplitude of the incident signals, and if one of the CMLs is replaced by a USPML with lattice constant of 0.5 mm, the peak value of the FEXT is $- 0.008$ V, i.e., the crosstalk accounts for about 4% of the amplitude of incident signals. The measurement result of the near-end crosstalk (NEXT) is presented in Fig. 8(d), where for the two parallel conventional microstrip lines, the measurement result of NEXT at *t* = 1.0 ns is 0.00617 V. If one of the parallel conventional microstrip lines is replaced by the bilateral or unilateral periodic microstrip line whose lattice constant is *d* = 0.5 mm, the NEXT measurement result is 0.00475 V or 0.00375 V, respectively.

## Conclusion

In order to reduce the electromagnetic interference, many methods to isolate the crosstalks between two microstrip lines have been proposed in succession^1–4^. In this work, we continue to study this topic in an alternative way, where the periodic microstrip lines have been utilized to efficiently reduce the electromagnetic interference between the adjacent microstrip lines. In the theoretical part of the present work, an explicit equation for the characteristic impedance of lossless periodic transmission lines has been proposed by using an equivalent circuit model. Theoretical analysis shows that when the transmission time of a step-function signal propagating through a unit cell of a periodic structure is less than half of the rise time of the step-function signal, the reflected wave of the step-function signal in the periodic structure will be covered by the rise time, and so the instantaneous impedance corresponding to the step signal detecting the periodic microstrip line is an average effect. By adjusting the geometric sizes of the periodic microstrip lines, the same instantaneous impedance as that of the conventional microstrip line can be obtained. In this way, it is possible to “deceive” the digital signals so that they cannot distinguish between the conventional microstrip lines and the present periodic microstrip lines. For the high-speed circuit systems, we expect that the conventional microstrip lines could be replaced completely by the present periodic microstrip lines when the relevant parameter conditions are met. In our work, the feasibility of such an idea has been fully verified by our actual circuit measurements.

## Methods

In our work, the circuit parameters and impedance of periodic microstrip lines were calculated by commercial FEM software (COMSOL). The time domain reflectometer was used to measure the instantaneous impedance of the periodic microstrip lines with different lattice constants, where the measured results have been compared with our numerical results.

## Data Availability

Data underlying the results presented in this paper are not publicly available at present but may be obtained from the corresponding authors upon reasonable request.
